# Cardiovascular manifestations in mast cell activation disease: key insights for cardiologists and angiologists

**DOI:** 10.3389/fcvm.2025.1705201

**Published:** 2025-11-10

**Authors:** Wolfgang Taumann, Gerhard J. Molderings

**Affiliations:** 1 Mast Cell Sciences Ltd., Meckenheim, Germany; 2 Institute of Human Genetics, University Hospital of Bonn, Bonn, Germany

**Keywords:** mast cell activation disease, systemic mastocytosis, mast cell activation syndrome, mast cell mediator release syndrome, mast cell mediators, cardiovascular symptoms

## Abstract

Mast cell activation disease is a genetic disease entity characterized by a very pronounced clinical symptomatology with potential manifestations in virtually every organ and tissue. These arise from the inappropriate release of mast cell mediators and the accumulation of both morphologically normal and mutated mast cells. Owing to the high prevalence of the disease—estimated to be up to 17%—cardiologists and angiologists are frequently confronted with mast cell activation disease in their daily clinical practice, often without recognizing it. Therefore, every cardiologist and angiologist should possess basic knowledge about this disease and be aware of its cardiovascular challenges. This review summarizes the current state of knowledge on this highly complex disease, with a particular focus on the cardiovascular aspects.

## 1 Introduction to mast cell activation disease

Mast cell activation disease (MCAD; Table 1) is a genetic disease entity with a very pronounced clinical symptomatology. Clinical manifestations can be present in potentially every organ and tissue. The clinical picture of MCAD is characterized in the majority of cases by pathologically increased mast cell activity resulting in an inappropriate release of mast cell mediators, accompanied by the accumulation of both morphologically normal and mutated mast cells in one organ, organ segment, or multiple organs in the proven absence of other underlying diseases. Although a procedure for diagnosing MCAD has been established (see Section 1.2), it takes, on average, more than 5 years (1) for a definitive diagnosis. The prevalence of the MCAD variant *mast cell activation syndrome* (MCAS; Table 1) in the Northern Hemisphere is estimated at 4%–17% (2, 3). Consequently, people with mast cell disease are not only frequently seen in primary care but also in cardiology and angiology patient populations and in emergency rooms. Hence, cardiologists and angiologists are repeatedly, often unknowingly, confronted with patients with mast cell activation disease. Moreover, in patients with MCAD, many guideline-recommended cardiovascular therapies have limitations. Various drugs are (relatively) contraindicated, poorly tolerated, or their efficacy is poorly documented due to a lack of studies. This review, therefore, focuses on the dual challenge of MCAD and cardiovascular disease in everyday clinical practice. Its aim is to support the cardiovascular care of patients with MCAD, since it is not only stressful but also life-threatening for patients with MCAD when mast cell disease-specific characteristics are not taken into account (4). The presented cardiovascular findings and their recommended treatment are based on an extensive literature search, consensus recommendations from various international expert groups (5, 6), the WHO recommendations for the MCAD variant “systemic mastocytosis,” and our own extensive experience in the treatment of patients with MCAD.

**Table 1:** Current classification of systemic forms of primary mast cell activation disease (modified from (5)).

Mast cell activation disease	
Systemic mastocytosis (SM)	Mast cell activation syndrome (MCAS)
Prevalence: 1–5/10,000	Prevalence: up to 17%
Clinical variants	
Indolent SM Well-differentiated SM Smoldering SM Aggressive SM SM with an associated hematological neoplasm Mast cell leukemia	Irritable bowel syndrome-phenotype Fibromyalgia-phenotype Cardiac phenotype CNS-phenotype Phenotype with anaphylactic reactions Mixed phenotype

Primary mast cell disease must be distinguished from secondary (i.e., non-genetic) mast cell activation (often abbreviated to MCA), which occurs in a variety of clinical contexts and pathologies, including IgE-dependent allergic inflammation and other immunological and inflammatory reactions.

### 1.1 Pathogenesis of MCAD

MCAD, regardless of its various manifestations, represents a multifactorial, polygenic, and, thus, primary disease entity, i.e., the affected mast cell is mutationally altered (7), inducing pathologically increased activity. Due to the increased activity of affected mast cells, MCAD is characterized by an inappropriate exocytotic release of potentially more than 390 mast cell mediators stored in granules (8). These include pre-stored mediators, such as histamine, TNFα, and tryptase; numerous *de novo*-synthesized lipid mediators, including eicosanoids, chemokines, and cytokines; and many others (8). In addition to this exocytotic release, mediators can be released or transferred via several other mechanisms [reviewed in (8)]; under certain circumstances, almost all molecules formed by a mast cell can function as mediators. Due to the ubiquitous distribution of mast cells in the organism, symptoms can occur as a result of temporary dysfunctions in virtually all body systems, i.e., organs and tissues, including the cardiovascular system [for a comprehensive list of symptoms in the potential affected systems, see (8)]. In a few patients, all systems are affected, with the majority displaying subsets. The majority of symptoms in the affected systems are chronic and low-grade; some are persistent, but many are either episodic or waxing/waning (9). Depending on the type and quantitative extent of mast cell mediator exocytosis and the compensatory mechanisms inherent in all tissues to eliminate mast cell mediators and their functional effects, the severity of the clinical picture can range from trivial to disabling and even life-threatening (10). The multisystem combination of these symptoms is referred to as *mast cell mediator release syndrome*.

### 1.2 How to diagnose mast cell activation disease?

While in many diseases, a suspected diagnosis is suggested based on certain key symptoms, this is not the case in MCAD. Patients with MCAD usually describe the diverse clinical symptoms of mast cell mediator release syndrome. It is not uncommon to find a history of previous doctor hopping (recognizable by the bulging file of medical reports and findings that is usually brought to the consultation), with a multitude of individual diagnoses (especially functional bowel disorders, histamine intolerance, salicylate intolerance, multiple allergies, and multiple drug intolerances) that alone cannot explain the overall clinical picture and for which specific therapies have been unsuccessful. Such constellations should raise suspicion of MCAD. Using a standardized, validated medical history checklist (2, 11, 12), the suspicion of mast cell mediator release syndrome can be substantiated early on. A point score is used to analyze whether a patient's symptom constellation can be attributed with a probability of 95% to an unregulated increased release of mast cell mediators. The objection occasionally raised against the medical history checklist, i.e., that the symptoms and findings listed therein are largely non-specific and could be triggered by a variety of diseases, is insufficient. It must be countered that, of course, the relevant differential diagnoses must be excluded. Ultimately, however, the decisive factor in diagnosing mast cell mediator release syndrome is the fact that these symptoms and findings, which are non-specific in themselves, occur in a combination that affects multiple body systems simultaneously. In analogous checklists from other international working groups [e.g., (13)], the involvement of three body systems is considered sufficient to confirm the suspicion of mast cell mediator release syndrome. Our current version of the checklist is more restrictive. Only when five body systems are affected does the score suggest suspicion with a probability of 95% (12). Whether MCAD is the underlying cause of the mast cell mediator release syndrome must then be proven using diagnostic criteria [details in (10, 14, 15)].

### 1.3 Cursory overview of MCAD therapy

Due to its genetic causation, this disease is incurable in the current state of medical science. Once the disease has manifested clinically, the symptoms usually progress at varying rates if left untreated. Variables influencing and modifying the disease process may include other genetic predispositions (e.g., Ehlers–Danlos syndrome, IL-4R polymorphisms, and diamine oxidase and histamine N-methyltransferase polymorphisms), environmental factors such as diet and lifestyle, immunoglobulin levels and function, microbiome, the number and type of infections, or vitamin D metabolism. The totality of these variables influences the severity and progression of this disease. Therefore, a complex therapy is necessary after diagnosis [Table 2; for review (16)]. An essential prerequisite for drug therapy is that the affected patients adapt their lifestyle to their disease and minimize the frequency of triggering mast cell activation. If this does not happen, the therapy cannot be satisfactorily successful because mast cell activation repeatedly counteracts the mast cell-inhibiting effect of the medication. This also includes avoiding foods containing gluten, cow's milk protein, and baker's yeast for several weeks before or at the latest upon initiation of drug therapy, as these can weaken the mast cell-calming effect of the medication through non-specific histamine release (e.g., by wheat lectins and casomorphins) and/or stimulation of the immune system in the intestine (17). Similar effects can also be induced by alcohol or its metabolite, acetaldehyde, and by salicylates in medications and cosmetics (16). Due to the highly individualized genetic distortion of mast cells, only personalized therapy can be successful, which must be continually adapted to an individual’s needs in terms of composition and dosage over the course of the disease.

**Table 2:** Drug treatment for MCAD (16).

First-line long-term therapy (therapeutic aim: reduction of the unregulated, pathologically increased activity of mast cells)
Sustained-release ascorbic acid, H_1_-anthistamine, H_2_-anthistamine, cromoglicic acid, ketotifen (as an alternative to cromoglicic acid in cases of intolerance or to extend therapy), montelukast (a cysteinyl leukotriene receptor 1 antagonist), medicinal *C. sativa* buds, omalizumab (anti-IgE-antibody), and flavonoids (e.g., quercetin and luteolin)
More incisive additional drugs for long-term therapy (therapeutic aim: inhibition of mast cell proliferation and survival)
Interferon-α, imatinib, avrapitinib, and midostaurin
Optional symptom-oriented therapy

In summary, the current first-line therapy consists of a long-term therapy consisting of an individually tailored combination of medications with sustained-release vitamin C, H_1_-antihistamine, H_2_-antihistamine, cromoglicic acid and/or ketotifen, montelukast, and if necessary, inhalation of medicinal *Cannabis sativa* buds [details in (16, 18)], aiming at reducing the extent of activation of mast cells and, depending on the active compound (antihistamines and montelukast), antagonizing their mediators at other mast cells and other immune cell populations (e.g., eosinophil granulocytes and dendritic cells), and preventing a secondarily induced pro-inflammatory state. Administered simultaneously with the mast cell activity-inhibiting therapy, symptom-oriented drug treatment should reduce the effects of the released mast cell mediators on effector cells by blocking the corresponding receptors on the effector cells, thus alleviating symptoms and preventing the establishment of a vicious circle. If this medication fails to achieve a tolerable disease intensity within a period of up to 3 months, it is indicated to escalate treatment to more profound therapeutics, such as immunosuppressive steroids (short-term use only), the anti-IgE antibody omalizumab, the anti-IL4/13 antibody dupilumab, kinase inhibitors (e.g., imatinib or avapritinib), or interferon-α. Their effects are based on the inhibition of mast cell proliferation and survival. A comprehensive, detailed description of treatment options is given in (16).

## 2 Cardiovascular symptoms of MCAD

The presence of mast cells has been documented in the human heart (19, 20, 21, 22). Mast cells reside in the interstitial space between the cardiomyocytes and are present in the endocardium, myocardium, and epicardium [for a review, see, e.g., (23, 24)]. Mast cells are also found around human coronary microvessels (23), in atherosclerotic plaques (25, 26), and often in close proximity to sensory nerves (22, 27, 94). Given the high density of mast cells in the cardiovascular system, up to 80% of patients with MCAD report cardiovascular symptoms (10, 28, 29, 30, 31, 32). However, there are currently no systematic studies on the occurrence of cardiovascular symptoms in MCAD; thus, in the presence of cardiovascular symptoms without a definite MCAD diagnosis, this diagnosis is rarely considered as the cause of the symptoms. The cardiovascular manifestations of mast cell activation linked to suspected causative mast cell mediators are summarized in Table 3.

**Table 3:** Cardiovascular symptoms of mast cell activation disease induced by mediators proven to be involved in the pathogenesis of the listed symptoms and dysfunctions (8, 47, 58, 76, 85, 89, 90, 91, 92, 93). The list is not exhaustive because there are probably more as yet unidentified mediators involved in the pathogenesis of the symptoms.

Cardiovascular symptoms	Mediators
Cardiac symptoms	
Dysrhythmias (sinus tachycardia, ventricular tachycardia, bradycardia, and supraventricular and ventricular extrasystoles)	Histamine, PGD_2_, MMPs, endothelin 1 and 3, adenosine, triiodothyronine, 3-iodothyroacetic acid; 3-iodothyroanamine, and platelet-derived growth factor-A
Palpitations	Not yet investigated
Cardiac arrest	Histamine, PGD_2_, and leukotriens
Pericarditis	Not yet investigated
Heart failure (classic and atypical heart failure, e.g., takotsubo cardiomyopathy)	Histamine, tryptase, chymase, platelet activating factor, renin, IL-4 and -6, TNFα, fibroblast growth factor-2 and -7, and TGF-ß
Cardiac fibrosis	Tryptase, chymase, TGF-ß, and TNFα
Myocardial remodeling	TNFα, MMPs, platelet-derived growth factor α, ANGII, TGF-ß, IL-10 and -13, chymase, tryptase, histamine, CXCL10, and vascular endothelial growth factor α
Ischemia-reperfusion injury	TNFα, tryptase, ANGII, and histamine
Blood pressure anomalies	
Hypotension with presyncope or syncope	Histamine; tryptase; chymase; phospho­lipases; heparin; vasoactive intestinal polypeptide; PGD_2_; PGE_2_; platelet-activating factor; IL-6; nitric oxide; adrenomedullin, ACE 2; kallikrein 1; kallikrein-related peptidases 2, 8, and 9; kininogen 1; and natriuretic peptide A
Arterial hypertension	Histamine, chymase, carboxypeptidase A, renin, endothelin 1 and 3, PGD_2_ by metabolite, thromboxane A_2_, leukotriens, ANGII, angiotensinogen, and peptide YY
Vascular symptoms	
Arterial atherosclerosis	Tryptase; chymase; histamine; heparin; IL-4, -6, -8, and -1ß; IFNγ; CCL2; CCL3; CCL4; CCL-5; TNFα; vascular endothelial growth factor; fibroblast growth factor-2 and -7, biglycan; and apolipoprotein E
Restenosis	Chymase, tryptase, TGF-β, and histamine
Vein graft hyperplasia	Histamine and chymase
Aneurysm/artery dissection	Chymase; tryptase; cathepsin G, MMPs; IL-6, -8, and -1ß; IFNγ; CCL2; CCL-5; and adrenomedullin
Raynaud´s phenomenon	Histamine, serotonin, renin, ANGII, angiotensinogen, endothelin 1 and 3, PGD_2_ by metabolite, chymase, thromboxane A_2,_ leukotriens, IL-6 and -8, and peptide YY
Vasculitis	Not yet investigated
Coronary syndromes with or without myocardial infarction (associated with rupture of an atherosclerotic plaque, partial or complete thrombosis, arterial embolism, and/or allergic coronary spasm)	ANGII, angiotensinogen, histamine, serotonin, IL-6, -8, CCL-5, endothelin 1 and 3, and leukotriens
Angioneogenesis	Angiogenin; CXCL12; CCL2; IL-8; platelet-derived growth factor; TNFα; IL-1β; IL-6; vascular endothelial growth factor; fibroblast growth factor-2; IFNγ; heparin; MMP-2, -9, -and 13; and angiopoetin-2
Aberrant angiogenesis (hemangiomas, arteriovenous malformations, and telangiectasias)	Mediators involved in angioneogenesis are assumed but not proven to be involved
Livedo reticularis	Not yet investigated
Hemorrhoids	Not yet investigated
Varicosities	TGF-ß
Flush	Histamine and PGD_2_
Hot flashes	Mediator-induced neural-mediated mechanis­ms may be involved
Clotting and bleeding abnormalities	
Tendency to bleed due to heparin release and hyperfibrinolysis	Heparin, vasoactive intestinal polypeptide, prostanoids, tryptase, chymase, tissue-type plasminogen activator, and urokinase
Thrombophilia	Histamine, factor VIII, and tryptase

## 3 Selected cardiovascular symptoms, dysfunctions, and therapeutic challenges in MCAD

The following topics were selected for detailed discussion because they frequently occur in patients with MCAD and because at least some of these mediators responsible for the symptoms and dysfunctions have been identified. Moreover, the therapeutic options can be defined with a certain degree of specificity.

### 3.1 Cardiac fibrosis

**Figure 1:**
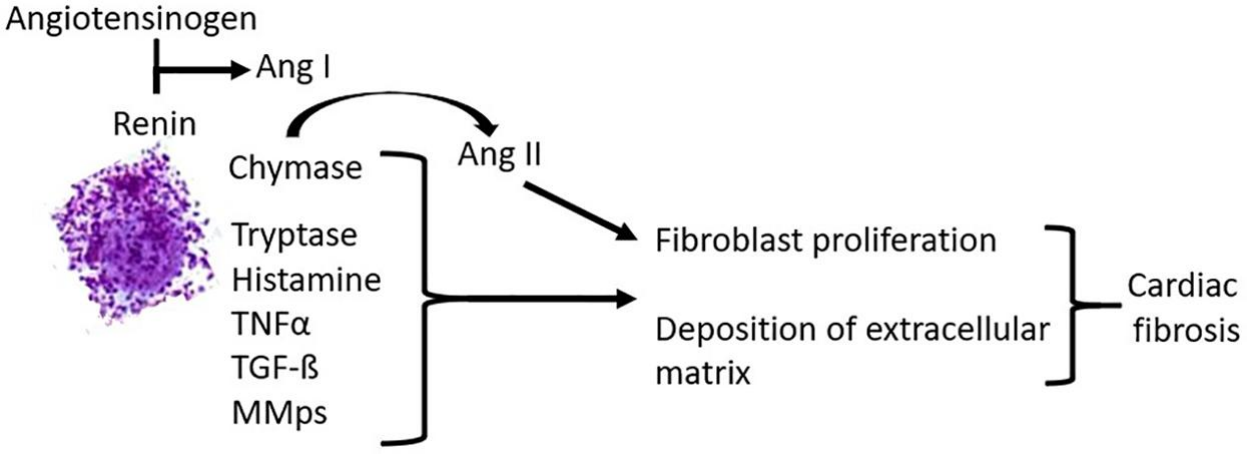
Pathogenetic effects of mast cells in cardiac fibrosis.

Experimental and *in vivo* evidence suggests a critical role for an increase in myocardial mast cell density in the pathogenesis of cardiac fibrosis by inducing the expansion of activated myofibroblasts. Activated cardiac mast cells release a variety of potent pro-inflammatory and pro-fibrotic mediators (Table 3), which may contribute to cardiac fibrosis [(33, 34); Figure 1]. Mast cell-derived proteases participate in matrix metabolism through fibrogenic mediators and matricellular proteins or by exerting contact-dependent actions on fibroblast phenotype. Mast cell chymase converts angiotensin I to angiotensin II (ANGII) independently of angiotensin-converting enzyme (ACE). ANGII generation directly contributes to fibrosis by inducing the differentiation of fibroblasts to myofibroblasts. Mast cell tryptase can act directly on fibroblasts to induce proliferation and differentiation to the myofibroblast phenotype. Tryptase and chymase both act on transforming growth factor-ß (TGF-ß) to convert it to the active form, which also induces fibroblast differentiation to the myofibroblast phenotype and collagen deposition. Mast cell-derived tumor necrosis factor α (TNFα) and interleukin 1ß (IL-1ß) induce cardiomyocyte apoptosis, MMP-9 production, and inflammatory cell recruitment that enhances tissue remodeling. Additionally, mast cells release TGF-ß, further contributing to the activation and differentiation of fibroblasts. Cardiac fibrosis is associated with systolic and diastolic dysfunction, arrhythmogenesis, and adverse outcomes.

#### 3.1.1 Therapeutic option

A blockade of mast cell activity using mast cell stabilizing drugs (Table 2) should be considered as a first-line therapy.

### 3.2 Dysrhythmias: sinus tachycardia, ventricular tachycardia, bradycardia, supraventricular and ventricular extrasystoles, and cardiac arrest

Dysrhythmias are frequently reported by patients with both MCAD variants (Table 1) (SM: 29% (35, 36); MCAS: ≥20% (29, 37, 38, 39, 40, 41)). In the worst case, MCAD can present as a cardiac emergency with cardiac arrest or ventricular fibrillation (42).

#### 3.2.1 Sinus tachycardia

Mast cell activation can induce an increased release of norepinephrine (NE) from cardiac sympathetic nerves, leading to pathological amounts of NE resulting in arrhythmias and sudden cardiac death. Mast cells can increase NE release by targeting sympathetic nerves in two ways. First, ANGII can be formed in the heart locally by mast cell-derived renin [for review, see (22)] or independently of ACE by the proteases cathepsin-D (43) and chymase [e.g., (44)]. ANGII then binds to facilitatory presynaptic angiotensin AT_1_-receptors on the sympathetic nerve endings, leading to presynaptic release of NE (45). In addition, ANGII can also directly elicit cardiac arrhythmias without sympathetic involvement (46). Second, mast cell-derived histamine is known to act as a direct stimulator of histamine-H_2_-receptors on the cardiomyocytes in the sinoatrial node (47), inducing an increase in heart rate and contractility in animal models and in humans (48) to elicit sinus tachycardia [for review, see (49)]. Finally, prostaglandin D_2_, which is also a major mast cell mediator, has been shown to induce tachycardia in humans (50).

##### 3.2.1.1 Therapeutic options

Mast cell mediator-induced supraventricular tachycardia should be treated when it causes extreme distress to the patient and when their heart rate persistently exceeds 100–120 beats per min. As a first step, the doses of drugs that reduce mast cell activity (Table 2), such as antihistamines (51), should be optimized. If this action is not sufficient to normalize the patient’s heart rate, further medical treatment should be considered. As a result of its pathophysiology, supraventricular tachycardia due to mast cell activation should be sensitive to treatment with direct renin inhibitors and AT_1_ receptor blockers. However, these drug classes can only be used in patients who do not have hypotension, since these drugs can induce a further decrease in blood pressure. In these patients, the funny current blocker ivabradine (52), which reduces heart rate in patients with sinus rhythm (but not in those with atrial fibrillation) without altering blood pressure (53), should be used next if available [as in Europe (14, 54)], since the calcium channel blocker verapamil is frequently ineffective (14). The use of ß-adrenoceptor antagonists should be avoided in patients with MCAD if possible. ß-Adrenoceptors play an important inhibitory role in the control of mast cell mediator release (55, 56). Hence, ß-adrenoceptor blockade disinhibits mast cell mediator release, usually leading to instantaneous aggravation of the symptoms of MCAD (57). If the use of ß-adrenoceptor antagonists is unavoidable, attempts must be made to block mast cell activity in patients with MCAD in parallel, e.g., by administering high doses of glucocorticoids. Finally, it should be noted that epinephrine, as an emergency medication to control anaphylactoid reactions, should be used in tachycardic patients with MCAD with particular caution because it has been reported to precipitate ventricular fibrillation (40). The antagonists of the two prostaglandin D_2_ receptors, *prostaglandin D_2_ receptor 1* (DP_1_) and *chemoattractant homologous receptor expressed on Th2 cells* (CRTH_2_), may theoretically be a future approach to the treatment of tachycardia in MCAD. However, clinical approval of such drugs is delayed because of safety concerns.

#### 3.2.2 Ventricular tachycardia

Ventricular tachycardia (VT) episodes in MCAD may only last a few seconds without causing harm, but episodes lasting more than a few seconds can be life-threatening. VT may sometimes result in ventricular fibrillation and turn into cardiac arrest (55). Pathophysiologically, the presumed causes of VT in association with MCAD include reentry circuits based on mast cell mediator-induced fibrosis and other myocardial tissue damage, as well as mast cell mediators, which are yet to be identified. Due to the above-discussed pathologically increased release of NE from cardiac sympathetic nerves, catecholaminergic polymorphic VT may occur.

##### 3.2.2.1 Therapeutic options

Therapy may be directed either at terminating an episode of abnormal heart rhythm or at reducing the risk of VT episodes. Usually, MCAD-related VT is self-limiting. Long-term anti-arrhythmic therapy may be indicated to prevent recurrence of VT. In several patients with MCAD, catheter ablation has been performed as a potentially definitive treatment option for their recurrent VT. However, probably due to the complex pathophysiology of VT in these patients with MCAD, VT reappeared after a few weeks or months (personal observation).

#### 3.2.3 Supraventricular and ventricular extrasystoles

Supraventricular (SVES) and ventricular extrasystoles (VES) are often found in patients with MCAD, in particular during flares of the disease but also after long-standing disease. The release of arrhythmogenic mast cell mediators is suspected to be the cause. The indication for drug therapy must be highly personalized.

#### 3.2.4 Bradyarrhythmia

The most prominent effect of histamine on the atrioventricular (AV) node is a slowing of conduction [for review, see (49)] up to a third-degree atrioventricular block (48, 59). In addition to the effect of histamine on the AV node, fibrosis of the AV node region can occur during the course of MCAD progression, resulting in a progressive AV block beginning with a first-degree AV block, transitioning into a second-degree AV block according to Mobitz type I or II, and evolving into a third-degree AV block. The progression of the AV conduction disturbance may manifest clinically as standing vertigo, coordination difficulties, and a rapid decrease in resting heart rate after physical exertion to values below 50–40 beats per min and sometimes even slower. A rapid decrease in heart rate may cause a drop in systolic blood pressure to 100 mm Hg or less, necessitating a temporary shock position. This physical stress reaction induces additional activation of the MCAD with a release of cardiovascular mediators, which can lead to a further drop in blood pressure (rarely to hypertension) and/or SVES and/or VES.

##### 3.2.4.1 Therapeutic option

Since the AV block in MCAD is not due to a temporary or reversible cause, the implantation of a dual-chamber pacemaker is indicated for any form of AV block of second degree or higher. After the implantation of the pacemaker, a temporarily rapid junctional escape rhythm and, in particular, SVES and VES may be observed due to released mast cell mediators. Since ß-adrenoceptor blockers should be avoided in MCAD (see Section 3.2.1), an individualized decision must be made as to whether and, if so, which antiarrhythmics should be used to suppress the dysrhythmias that are not eliminated by the pacemaker.

### 3.3 Heart failure

A role of mast cells in heart failure has been suggested [e.g., (60)]. In a Danish retrospective cohort study of 548 adults with systemic mastocytosis, 12 patients had congestive heart failure (61). In a prospective study with 18 patients with MCAS that was performed to investigate the suspected cardiac impact of increased systemic mast cell activation, diastolic left ventricular dysfunction was found using pulse wave- and/or tissue-Doppler imaging in 12 patients with MCAS, which represents the most sensitive sign of a myocardial structural alteration (62, 63). In five of these 12 patients, left ventricular hypertrophy was observed. Although there may be structural signs of heart failure in the majority of patients with MCAD, symptomatic chronic heart failure does not appear to be more prevalent than in the general population.

#### 3.3.1 Role of mast cells in pathogenesis

Alterations in the pumping performance of the heart in patients with MCAD are probably due to fibrosis and to the remodeling effect of the prohypertrophic and profibrotic factors secreted by mast cells (Table 3, Figure 1).

#### 3.3.2 Therapeutic options

The medical treatment options for patients with chronic heart failure [New York Heart Association (NYHA) class II–IV], based on the Guidelines of the European Society of Cardiology with a special focus on heart failure (64), are also valid for patients with MCAD and clinically manifest heart failure. The European Society of Cardiology Guidelines recommend, depending on the distortion of the ejection fraction, therapy with diuretics and SGLT-2 inhibitors, followed by the administration of angiotensin-AT_1_-receptor antagonists, ACE inhibitors, angiotensin receptor-neprilysin inhibitors, mineral corticoid receptor antagonists, and ß-adrenoceptor blockers. However, ACE inhibitors should be avoided in patients with MCAD if possible because they can significantly worsen MCAD: ACE is able to degrade bradykinin, which is an activator of mast cells (57). Therefore, the inhibition of ACE increases mast cell mediator releasability and, hence, autocrine mast cell activation with MCAD symptoms through the stabilization of bradykinin (57). ß-Adrenoceptor blockers should be used with great caution in patients with MCAD, as substantiated in Section 3.2.1. The use of SGLT-2 inhibitors should be considered in MCAD, as they could also possibly have a beneficial effect on MCAD itself. Since allergic reactions (e.g., urticaria) are possible, individual risk assessment and close monitoring in patients with MCAD are advisable, especially during the introduction phase of SGLT-2 inhibitors.

### 3.4 Hypotension with or without presyncope or syncope

Episodes of hypotension with lightheadedness or syncope as a manifestation of MCAD are reported by 22%–55% of the patients with MCAD (2, 29, 35, 36, 65), in contrast to a prevalence of 5% in the control group (2). The frequency of the episodes of syncope can vary from as frequently as daily to as rarely as once per year or never.

#### 3.4.1 Role of mast cells in the pathogenesis

Mast cells can produce and release a number of mediators that induce vasodilatation (Table 3) and, thereby, induce hypotension up to vascular collapse.

#### 3.4.2 Therapeutic options

In a scenario of acute hypotension, the simplest treatment is to lie back down immediately after feeling lightheaded upon standing. Symptoms will often disappear. In order to prevent mast cell mediator-induced hypotension, mast cell activity stabilizing drugs (Table 2), such as H_1_-antihistamines, should be the first-line therapy. Acetylsalicylic acid (80–325 mg orally once or twice daily in adults) may be beneficial in some patients who have high levels of vasodilating prostaglandins (36, 66, 67) if the patient's tolerance to use non-steroidal anti-inflammatory drugs (NSAIDs) without adverse effects is known. Alternatively, prostaglandin formation can be reduced through the selective inhibition of cycloxygenase-2, such as with etoricoxib in Europe or celecoxib in the US, though the potential cardiovascular risks of cycloxygenase-2-selective NSAIDs need to be acknowledged (68). Preliminary data from SM patients suggest the efficacy of the humanized monoclonal IgE-antibody omalizumab in the prevention of spontaneous episodes of systemic hypotension (69, 70, 71, 72, 73). In the case of recurrent anaphylactic syncope, acute treatment with epinephrine and corticosteroids should be considered. A future approach to the treatment of vascular symptoms in MCAD could be antagonists of the prostaglandin D_2_ receptors DP_1_ and CRTH_2_. However, no substances with these two targets have been approved thus far.

### 3.5 Systemic arterial hypertension

In up to 31% of patients with MCAD, marked recurrent or sustained elevation in arterial blood pressure due to mast cell activation has been observed (2, 29, 30, 74). Moreover, in patients with MCAD, alternating hypotensive and hypertensive episodes are often observed.

#### 3.5.1 Role of mast cells in the pathogenesis

Mast cells can produce and release a number of mediators that induce vasoconstriction (Table 3) and, thereby, induce hypertension. In particular, ANGII is a potent vasoconstrictor. Another major mediator released in patients with MCAD, the vasodilator prostaglandin D_2_, can be metabolized by 11-ketoreductase to the biologically active metabolite 9α, 11ß-PGF_2_, which is a vasopressor (74). Thus, arterial hypertension in patients with MCAD may be linked to the metabolism of prostaglandin D_2_ in the individual patient. In addition, released endothelins could be important mediators in the induction of arterial hypertension.

#### 3.5.2 Therapeutic options

As a result of its pathophysiology, therapy for hypertension in patients with MCAD must be personalized and is frequently developed through trial and error. Theoretically, MCAD-induced hypertension may be especially sensitive to treatment with direct renin inhibitors and AT_1_ receptor blockers. However, mast cell mediator-induced hypertension has often been shown to be refractory to these drugs. Calcium channel blockers such as amlodipine and, if these are insufficient, clonidine could be used. A blood pressure crisis due to an acute mast cell mediator release episode can be treated with nifedipine or clonidine, but in patients with MCAD, the blood pressure values may only decrease slowly over hours after administration. Nevertheless, the dosages of both drugs must be titrated very cautiously because the degradation of the causative hypertensive mast cell mediators can be shorter than the half-life of the drugs. If doses of nifedipine or clonidine are chosen that reduce the hypertension to normal blood pressure values, the drugs could induce dramatic hypotension within minutes when the hypertensive mediators are degraded. Therefore, in the interventional treatment for a hypertensive crisis in patients with MCAD, doses of both drugs should be chosen that will initially reduce the systolic blood pressure to an empirically found target value of 150–160 mmHg. As concentrations of hypertensive mast cell mediators in blood decrease, blood pressure will then continue to decline slowly over several hours, but will normally not reach extreme hypotensive levels. From the standard medication for the treatment of high blood pressure, ß-adrenoceptor antagonists and ACE inhibitors are problematic for the reasons mentioned above (Sections 3.2.1 and 3.3). The administration of endothelin receptor antagonists may be an approach to the treatment of arterial hypertension in patients with MCAD. In 2024, the US Federal Drug Administration approved the non-selective endothelin receptor antagonist aprocitentan for resistant hypertension. To date, there are no published studies on its use in patients with MCAD. In the future, if approved, antagonists of the prostaglandin D_2_ receptors DP_1_ and CRTH_2_ and peptide YY receptor antagonists, specifically targeting the Y1 and Y2 receptors, may become important drugs in the treatment of hypertension. In order to prevent mast cell mediator-induced hypertension, mast cell activity reducing therapy must be optimized (Table 2).

### 3.6 Arterial atherosclerosis

Atherosclerosis is a chronic inflammatory disease characterized by the formation of atherosclerotic plaques that consist of numerous cells, including smooth muscle cells, endothelial cells, immune cells, and foam cells. There is compelling evidence that mast cells play a pivotal role in the pathology of atherosclerosis [for a review, see, e.g., (75, 76)].

#### 3.6.1 Role of mast cells in pathogenesis

Mast cells accumulate within atherosclerotic plaques during their progression and release a mélange of mediators, including histamine, tryptase, chymase, cathepsins, and cytokines, such as TNFα, IFNγ, CCL2, IL-6, and IL-8. These mediators promote vascular cell apoptosis, blood-borne inflammatory cell adhesion and recruitment, foam cell formation, lipid degradation, neovascularization of the plaque, plaque progression, instability, erosion, rupture, aggravation of the inflammation of the plaque environment, and thrombosis (76, 77).

#### 3.6.2 Therapeutic options

The currently available therapeutics for atherosclerosis focus on alleviating hyperlipidemia and preventing thrombotic complications. The current first-line clinical drugs for lipid management are statins, which, in addition to their main effect of lowering cholesterol levels, have pleiotropic effects, e.g., reducing the activity of mast cells (16). Non-statin agents, including ezetimibe and proprotein convertase subtilisin/kexin type 9 (PCSK9) therapies, complement statin therapy or are administered independently. In addition, therapies that disrupt atheroprogression through immunosuppressive mechanisms have been investigated in clinical trials [reviewed in (78)]. In this context, the drug-induced reduction in mast cell activity in patients with MCAD is an important preventive anti-atherogenic therapy, since mast cells actively contribute to atherosclerotic plaque progression and destabilization (Table 2). Cholesterol and triglycerides that are diet-independently increased in the blood in approximately 75% of MCAD patients (28) can be deposited in the artery walls, forming plaques that narrow the vessels and reduce blood flow.

### 3.7 Angina pectoris and coronary syndromes with or without myocardial infarction

#### 3.7.1 Angina pectoris due to coronary heart disease

Angina pectoris can be the cardinal symptom of coronary artery disease, in which the underlying circulatory disorder is caused by stenosis of a coronary artery due to atherosclerosis. This form of angina pectoris responds to the administration of nitroglycerin spray, and the pain is aggravated by exertion in acute scenarios. The MCAD variant systemic mastocytosis has been proven to be associated with a higher incidence of acute coronary syndrome, even when plasma lipid levels are low (79).

#### 3.7.2 Vasospastic angina pectoris

Another cause of coronary artery narrowing in patients with MCAD is coronary artery vasospasm that can be detected acutely on an ECG by screening for signs of ischemia. However, the ECG changes are reversible and physical performance is good. Typically, these signs are no longer present on the ECG upon arrival at the emergency room, and troponin levels in the blood are not elevated. Furthermore, this form of angina pectoris responds to the administration of nitroglycerin spray, and the pain is aggravated by exertion in the acute scenario. Numerous case reports of patients with MCAD presenting with vasospastic angina pectoris have been published (31, 80, 81, 82, 83). We have made similar observations in our patient population (unpublished findings). Mast cells have been detected at the site of vasospasm in patients with variant angina, indicating a role in coronary artery spasm (19). Vasospastic angina pectoris in patients with MCAD seems to be due to the release of a plethora of vasoconstrictory mediators, such as serotonin, leukotriens, and endothelin, from mast cells (Table 3), which can trigger coronary artery vasospasm.

*Therapeutic options for 3.7.1 and 3.7.2*

In patients with MCAD, the treatment should initially dilate the coronary vessels in both variants. Vasodilator drugs, including nitrates, e.g., nitroglycerin and calcium channel blockers, should be considered as the first-line therapy. For the vasospastic variant, after initial relief of vasospasm with a vasodilator drug, blockade of mast cell activity with mast cell stabilizers (Table 2) should be attempted to prevent coronary artery vasospasm. Such optimized, long-term mast cell activity-inhibiting medication, always adapted to the specific trigger situation, should be used for the prophylaxis of mast cell-induced vasospastic angina pectoris.

#### 3.7.3 Neuropathic non-cardiac angina pectoris

There are patients with MCAD whose angina pectoris does not respond to the application of vasodilators, is not triggered or worsened by exercise, and usually occurs spontaneously at rest. However, it can be alleviated by blocking mast cell activity. It is assumed that this angina pectoris reflects neuropathic pain resulting from the activation of mast cells. Mast cells are often in close proximity to sensory nerves (22, 27, 94), which may be relevant to the occurrence of non-cardiac angina pectoris. If C-nerve fibers in the thorax are activated by mast cell mediators, their activity is interpreted in the brain as angina pectoris-like. There are no systematic studies on the prevalence of neuropathic non-cardiac angina pectoris in patients with MCAD. However, non-cardiac angina pectoris is frequently observed in MCAD treatment centers. If the presence of MCAD is unknown in a patient, the frequency of rescue calls with admission of such a patient to the emergency room may be as high as once a week. In the absence of signs of ischemia on the ECG and pathologically altered specific blood values, as well as independence from exercise, emergency physicians should consider non-cardiac angina pectoris due to MCAD.

*Therapeutic options for 3.7.3*

Blockade of mast cell activity with mast cell activity-inhibiting drugs should be considered as the first-line therapy (Table 2).

#### 3.7.4 Non-cardiac angina pectoris due to Roemheld symptom

Mast cell mediator-induced adhesions can lead to an accumulation of gas before the upper left intestinal flexure. In addition, extreme abdominal distension often occurs unpredictably and within minutes, independent of food intake. Focal swelling, segmental or local hypo-, hyper-, and dysmotility, with transport disturbances, induced by acute mast cell mediator release, appear to be responsible for this phenomenon. This flatulence results in pain from adjacent organs, such as cardiac pain. The resulting displacement of the cardiac axis in cases of pronounced flatulence in the upper small intestine and stomach can lead to the Roemheld symptom complex (84).

*Therapeutic options for 3.7.4*

In such cases, the use of liquid simethicone, heat (placing a heating pad on the abdomen or drinking 500 mL of warm water), and, if indicated due to the intensity of the symptoms, the administration of metamizole as a spasmolytic (check tolerance beforehand), as well as intravenous administration of antihistamines and fluid resuscitation with 5% glucose or an electrolyte solution, may be helpful in the acute scenario (84). Preventive treatment focuses on optimizing MCAD medication (Table 2) and dietary changes, e.g., avoiding gas-producing foods.

### 3.8 Clotting and bleeding abnormalities

Mast cell activation may affect hemostasis through vascular (e.g., endothelial cells) and cellular components (e.g., platelets, monocytes, and neutrophils) and by clotting and fibrinolytic factors released from activated mast cells [for a review, see (85)]. Since high concentrations of mast cell mediators related to secondary hemostasis can be achieved in circulation, clinically significant bleeding, thrombosis, or both simultaneously can occur in MCAD. The resulting platelet activation may additionally trigger mast cell activation, thereby stimulating mast cell mediator release and contributing to the distortion of hemostasis. Clinical signs of bleeding diathesis, such as hematoma formation, bruising, prolonged bleeding after biopsies, gingival bleeding, epistaxis, gastrointestinal hemorrhage, conjunctival hemorrhage, menorrhagia, or hemorrhagic ulcer disease, occur in approximately 50% of patients with MCAD; severe or fatal bleeding seems to be rare (85). Increased release of heparin from mast cells (the main source of heparin) can reach circulating concentrations as high as the heparin levels achieved during thromboprophylaxis through subcutaneously applied exogenous heparin and, thereby, could contribute to bleeding diathesis. However, the main cause of bleeding diathesis likely lies in a serious hyperfibrinolysis. Therefore, in all patients suspected of having MCAD, a mast cell disease-specific examination of the coagulation system should be carried out for diagnostic reasons and before surgeries to optimize the perioperative procedures.

#### 3.8.1 Therapeutic options

To reduce thrombotic and bleeding tendencies in patients with MCAD in their everyday lives, mast cell activation should be reduced as much as possible by the class of medication found to be best for the individual patient (Table 2).

Hemostatic drug treatment for bleeding in patients with MCAD is only based on clinical evidence. Before and after invasive procedures, mast cell activity should be reduced as much as possible by administering medications and avoiding triggers such as temperature shock from a cold operating room or infusion of refrigerated fluids. Routine procedures to stop surgical bleeding are rarely effective when the bleeding is induced by mast cell activity. Unless contraindicated, tranexamic acid (TXA) 1 g should be administered intravenously 30 min before the first incision, and, depending on the intra- and postoperative bleeding situation, the TXA infusion should be continued for at least 12–24 h (total dosage 2–3 g/24 h). Depending on the clinical circumstances, TXA can be administered topically, orally, or intravenously. The risk of an arterial or venous thromboembolism when using TXA remains unclear but appears to be empirically low with the recommended dosages.

In cases in which bleeding is proven to be due to very high plasma heparin levels, heparin could be neutralized through protamine titration according to the patient's heparin levels. However, because of possible adverse reactions to protamine due to mast cell activation, protamine use should be limited to patients with MCAD with life-threatening bleeding, which is very likely the result of endogenous heparin.

In cases of severe thrombocytopenia (due to hypersplenism in an enlarged spleen, which is often present in MCAD, among other causes ), a transfusion of platelet concentrates should be considered.

Despite the potential risk of bleeding in patients with MCAD, thromboprophylaxis with low-molecular-weight heparin, unfractionated heparin, or fondaparinux should not be withheld when clinically indicated, including in the context of internal or surgical treatment.

Thrombophilia and a thromboembolism in patients with MCAD should be treated according to the respective guidelines.

### 3.9 Raynaud's phenomenon

Raynaud's phenomenon [RP; for review, see, e.g., (86)] is a condition characterized by episodic, excessive vasoconstriction triggered by cold or stress in the fingers and toes, albeit less pronounced in the latter. This leads to a distinctive sequence of color changes of the digits, which is usually livid in patients with MCAD. The majority of patients experience numbness and/or severe burning pain. Nailfold capillaroscopy may reveal reduced flow with marked sludge formation, episodes of stasis, and intermittent pendulum flow. Capillary density is often only slightly reduced without true avascular fields. Atypical capillaries, particularly torsions and vertex ectasias, may be present. Microbleeds may occasionally be observed. These changes may be accompanied by perivascular edema. Acral oscillography of the upper extremities shows reduced oscillations after cold exposure. Cold autoantibodies are not detectable in the blood. In the context of MCAD, the pathogenesis of RP presumably involves a complex interaction between the vascular wall, nerves, and mast cell mediators, disrupting the balance between vasoconstriction and vasodilation (Table 3). The prevalence of RP in MCAD has not been determined as yet. An estimation of up to 10% reflects our experiences.

#### 3.9.1 Therapeutic options

Although RP is suggested to be induced by mast cell mediators in MCAD, its treatment remains elusive. After its manifestation in MCAD, RP initially responds a little to mast cell activity-inhibiting drugs, but as MCAD progresses, it no longer responds at all. Patients are advised to avoid cold exposure. Otherwise, the recommended medication is that for RP management in general: vasodilators (dihydropyridine calcium channel blockers, topical nitrates, pentoxifylline, and phosphodiesterase-5 inhibitors), vasoconstriction inhibitors (ANGII receptor antagonists and endothelin-1 receptor antagonists), and endothelial function modulators (limited evidence and no strong recommendation for the use of antiplatelet agents). Studies on whether these drugs have a beneficial effect in MCAD-related RP are missing.

### 3.10 Cardiac catheterization and pacemaker implantation

The special precautions indicated for major surgical interventions in patients with MCAD (87) should also be applied to less invasive procedures, such as cardiac catheterization and pacemaker implantation. Both procedures should be performed under short-term anesthesia with propofol. Propofol is particularly suitable for this purpose because, in addition to its narcotic effect, it simultaneously inhibits mast cell activity. During these procedures, the tendency to bleed, which exists in almost all patients with MCAD, should be reduced through a preoperative administration of tranexamic acid (see Section 3.8).

Furthermore, it should be taken into account during pacemaker implantation that patients with mast cell disease have reduced ability to dissolve absorbable suture material (88). Therefore, absorbable suture material should not be used to prevent impaired postoperative wound healing. In addition, the implanted pacemaker should allow for MRI examinations with a magnetic flux density of 3 Tesla, as patients with MCAD must undergo repeated MRI scans due to their multisystem symptoms and comorbidities.

## 4 Limitations of this review

The significance of the above statements is limited because they are based only on small study groups, individual case reports, and the experience of MCAD experts. Although the majority of patients with MCAD complain of cardiovascular symptoms, no systematic studies have been conducted to date on the various cardiovascular symptoms. Nevertheless, there is no reason to doubt the causal involvement of mast cell mediators in the described symptoms in MCAD.

However, without results from systematic studies, no long-term prognostic data can be presented regarding the cardiovascular manifestations of MCAD, including mortality, and whether treatment interventions improve cardiovascular outcomes in MCAD.

Finally, most treatment recommendations are based on expert opinion, albeit obtained in the treatment of thousands of patients. Thus, there is a need for prospective randomized controlled trials.

## 5 Conclusion

Cardiologists and angiologists are often unknowingly confronted with mast cell activation disease in their daily clinical practice. Therefore, every cardiologist and angiologist should have a basic understanding of this disease and should be informed about its challenges in the cardiovascular system.
